# Dr. Wilbur F. Litch

**Published:** 1913-03-15

**Authors:** 


### OBITUARY.

---

Dr. Wilbur F. Litch. Dr Litch died at his home in Ardmore, Pa., December 25th, 1912, in the seventy-second year of his age.

At the time of his death Dr. Litch was Editor of the *Dental Brief*, which he had so ably edited for the past twelve years. His death was sudden and caused by heart failure induced by a severe attack of the Grippe.

Dr. Litch began the study of dentistry in 1858 in the office of a Philadelphia dentist and graduated from the Pennsylvania College of Dental Surgery in 1861 and in 1865 graduated in Medicine from Jefferson Medical College. He was appointed Professor of Dental Therapeutics and Prosthetic Dentistry in the Pennsylvania College of Dental Surgery in 1878, and became its Dean in 1899. He was a prolific writer and contributor to the current literature of his profession.

His great literary work was the American System of Dentistry, a three volume treatise on all phases of dental practice. Much of the material was contributed by a large number of authors, but three of the strongest chapters were written by Dr. Litch. This work took five years of his time to produce. He was a man of the highest ideas from every standpoint. As a teacher he exemplified these ideals. He was of a somewhat reserved manner, but a man of kindest disposition and always appreciative of goodness and earnestness of purpose in others. He was one of our great men.

